# Common genetic variants with fetal effects on birth weight are enriched for proximity to genes implicated in rare developmental disorders

**DOI:** 10.1093/hmg/ddab060

**Published:** 2021-03-04

**Authors:** Robin N Beaumont, Isabelle K Mayne, Rachel M Freathy, Caroline F Wright

## Abstract

Birth weight is an important factor in newborn survival; both low and high birth weights are associated with adverse later-life health outcomes. Genome-wide association studies (GWAS) have identified 190 loci associated with maternal or fetal effects on birth weight. Knowledge of the underlying causal genes is crucial to understand how these loci influence birth weight and the links between infant and adult morbidity. Numerous monogenic developmental syndromes are associated with birth weights at the extreme ends of the distribution. Genes implicated in those syndromes may provide valuable information to prioritize candidate genes at the GWAS loci. We examined the proximity of genes implicated in developmental disorders (DDs) to birth weight GWAS loci using simulations to test whether they fall disproportionately close to the GWAS loci. We found birth weight GWAS single nucleotide polymorphisms (SNPs) fall closer to such genes than expected both when the DD gene is the nearest gene to the birth weight SNP and also when examining all genes within 258 kb of the SNP. This enrichment was driven by genes causing monogenic DDs with dominant modes of inheritance. We found examples of SNPs in the intron of one gene marking plausible effects via different nearby genes, highlighting the closest gene to the SNP not necessarily being the functionally relevant gene. This is the first application of this approach to birth weight, which has helped identify GWAS loci likely to have direct fetal effects on birth weight, which could not previously be classified as fetal or maternal owing to insufficient statistical power.

## Introduction

Weight at birth is an important factor in newborn and infant survival (1), and it is associated with a higher risk of adverse adult health outcomes at both the high and low ends of the population distribution (2–4). Variation in birth weight is influenced by a combination of environmental and genetic factors, and genome-wide association studies (GWAS) of birth weight have implicated 190 genomic loci to date (5–7). The associated variants at three-quarters of the identified loci where classification is possible show direct effects of the fetal genotype, a small proportion of which also show maternal effects. The rest represent single nucleotide polymorphisms (SNPs) having only indirect effects of the maternal genotype (acting via the intrauterine environment) (5). Knowledge of the causal genes and biological pathways underlying birth weight variation will be crucial to understanding its links with infant and adult morbidity. However, causal variants at the identified GWAS loci have not yet been identified; many of the SNPs that mark the association signals fall outside coding regions, and it is unclear whether the functional variant they are tagging exerts its effect via the nearest gene or elsewhere.

Rare developmental syndromes arising from severe mutations in a single, known gene may provide valuable information to help prioritize candidate genes at the GWAS loci (8–10). Numerous monogenic developmental syndromes include either extreme fetal overgrowth (e.g. Cantu syndrome caused by mutations in *ABCC9* (11) and Clove syndrome caused by mutations in *PIK3CA* (12)) or severe fetal growth restriction (e.g. Floating-Harbor syndrome caused by mutations in *SRCAP* (13,14) and Myhre syndrome caused by mutations in *SMAD4* (15,16)). The overlap between genes with monogenic effects on birth weight and loci associated with birth weight from GWAS has not been formally examined.

**Figure 1:**
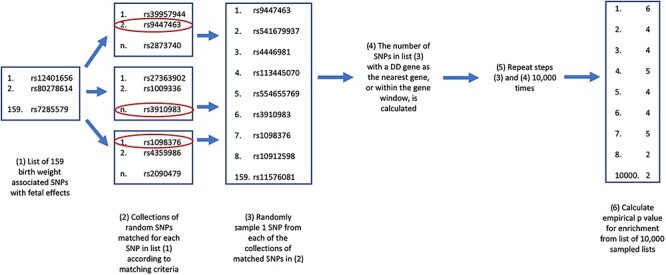
Flow chart showing each of the steps in the enrichment analysis.

Following a previous GWAS of adult height, Wood *et al*. (17) used a curated list of genes associated with rare human conditions of abnormal skeletal growth to investigate the identified loci. They hypothesized that common variation in or near the genes on the list would underlie several of the GWAS signals and thereby implicate biological pathways of relevance to normal variation in adult height. They found that the height GWAS loci were 1.4-fold more likely to fall near to the curated list of genes than simulated lists of randomly selected SNPs/indels. For fetal genetic variation underlying birth weight variation, it is not known whether a similar relationship exists between monogenic and polygenic loci. If such an overlap exists, it could help to prioritize candidate genes at these loci and to understand the biological pathways underlying birth weight. We tested whether the genes known to cause severe developmental disorders (DDs) (18) were nearer lead birth weight GWAS SNPs with evidence of fetal effects, than expected by chance (Fig. 1). Genes implicated in DDs were chosen because extremes of birth weight are frequently seen in DDs. To maximize power, we chose to include all genes implicated in DDs rather than limiting our analyses solely to DD genes with birth weight recorded as a feature of the associated disorder because many of the DDs are extremely rare and lack detailed phenotype information. By including all DD genes, we would capture those effects on birth weight which had not been recorded. We found evidence that the birth weight GWAS SNPs tested fell disproportionately close to genes that cause severe DDs and that this was driven by disease genes that act via a dominant mechanism. This approach helps to highlight potentially causal genes at GWAS loci, underscored by the fact that, for 24 of the 37 SNPs falling near to DD genes, the nearest gene to the SNP was not the DD gene.

## Results

### Method validation

#### Positive control: height SNPs

There was strong evidence of enrichment for DD genes being the closest gene to height SNPs (Tables 1 and 2). Of the 1362 DD genes, 81 were the closest gene to at least one height SNP (*P* < 0.0001), and 46 of these genes act in a dominant manner and 35 are recessive-only (*P* < 0.0001, *P* = 0.0002, respectively). From the 694 height SNPs, 97, 55 and 42 have a DD gene as the nearest gene from the full list of genes, list of dominant genes and genes with only recessive effects, respectively (*P* < 0.0001, *P* < 0.0001, *P* = 0.0011). These results mirror those of Wood *et al* (17) which found strong evidence of enrichment for genes underlying monogenic syndromes of abnormal skeletal growth. In the window analysis, dominant DD genes show consistent strong evidence for enrichment within the 19, 94, 138 and 258 kb windows (48 genes, *P* < 0.0001; 84 genes, *P* < 0.0001; 103 genes, *P* < 0.0001; 136 genes, *P* < 0.0001, respectively). There is only weak evidence that recessive-only genes are enriched within these windows (46 genes, *P* = 0.0013; 88 genes, *P* = 0.017; 124 genes, *P* = 0.0099; 185 genes, *P* = 0.010, respectively). The number of height SNPs with recessive-only DD genes within each window shows no evidence of enrichment (51 SNPs, *P* = 0.051; 94 SNPs, *P* = 1.00; 128 SNPs, *P* = 1.00; 180 SNPs, *P* = 1.0, respectively).

**Table 1:** Number of DD genes which are the nearest gene, or within the corresponding window, of 694 height SNPs, and the corresponding empirical *P*-value

Gene list (number of genes)	Nearest		19 kb		94 kb		138 kb		258 kb	
	*N*	*P*	*N*	*P*	*N*	*P*	*N*	*P*	*N*	*P*
All genes (1362)	81	<1.00E-4	94	<1.00E-4	172	4.50E-03	227	2.00E-04	321	4.60E-03
Dominant (475)	46	<1.00E-4	48	<1.00E-4	84	<1.00E-4	103	<1.00E-4	136	<1.00E-4
Recessive (936)	46	<1.00E-4	57	3.00E-04	105	1.15E-02	141	7.00E-03	208	9.80E-03
Recessive excluding dominant (887)	35	2.00E-04	46	1.30E-03	88	1.74E-02	124	9.90E-03	185	1.00E-02

**Table 2:** Number of height SNPs (total N_SNPs_ = 694) for which a DD gene is the nearest gene, or for which a DD gene is within the corresponding window, and the corresponding empirical *P*-value

Gene list (number of genes)	Nearest		19 kb		94 kb		138 kb		258 kb	
	*N*	*P*	*N*	*P*	*N*	*P*	*N*	*P*	*N*	*P*
All genes (1362)	97	<1.00E-4	103	<1.00E-4	177	2.69E-01	228	1.05E-01	287	9.62E-01
Dominant (475)	55	<1.00E-4	55	<1.00E-4	98	<1.00E-4	125	<1.00E-4	163	<1.00E-4
Recessive (936)	56	<1.00E-4	65	2.00E-04	115	8.83E-01	148	9.10E-01	202	1.00E+00
Recessive excluding dominant (887)	42	1.10E-03	51	5.12E-02	94	9.98E-01	128	9.97E-01	180	1.00E+00

#### Negative controls: eye color and random SNPs

As expected, SNPs associated with eye color show no evidence of enrichment for proximity to DD genes (all *P* > 0.05; Tables 3 and 4). The lack of enrichment in these SNPs suggests that the method is working as expected, however, the small number of SNPs associated with eye color could contribute to the lack of evidence for enrichment. We therefore also randomly selected 156 SNPs, which also showed no evidence for enrichment in either the nearest gene analysis or windows analysis (Tables 5 and 6).

**Table 3:** Number of DD genes which are the nearest gene, or within the corresponding window, of 16 eye color SNPs, and the corresponding empirical *P*-value

Gene list (number of genes)	Nearest		19 kb		94 kb		138 kb		258 kb	
	*N*	*P*	*N*	*P*	*N*	*P*	*N*	*P*	*N*	*P*
All genes (1362)	3	9.69E-02	3	1.00E-01	3	3.56E-01	3	4.62E-01	3	6.27E-01
Dominant (475)	1	3.81E-01	1	3.59E-01	1	5.36E-01	1	6.00E-01	1	7.08E-01
Recessive (936)	2	1.52E-01	2	1.68E-01	2	4.26E-01	2	4.99E-01	2	6.37E-01
Recessive excluding dominant (887)	2	1.33E-01	2	1.43E-01	2	3.95E-01	2	4.71E-01	2	6.10E-01

**Table 4:** Number of eye color SNPs (total N_SNPs_ = 16) for which a DD gene is the nearest gene, or for which a DD gene is within the corresponding window, and the corresponding empirical *P*-value

Gene list (number of genes)	Nearest		19 kb		94 kb		138 kb		258 kb	
	*N*	*P*	*N*	*P*	*N*	*P*	*N*	*P*	*N*	*P*
All genes (1362)	3	1.47E-01	3	1.28E-01	3	4.21E-01	3	5.50E-01	3	7.77E-01
Dominant (475)	1	4.44E-01	1	4.05E-01	1	6.00E-01	1	6.72E-01	1	8.19E-01
Recessive (936)	2	2.03E-01	2	2.04E-01	2	4.97E-01	2	5.88E-01	2	7.80E-01
Recessive excluding dominant (887)	2	1.81E-01	2	1.79E-01	2	4.63E-01	2	5.60E-01	2	7.52E-01

**Table 5:** Number of DD genes which are the nearest gene, or within the corresponding window, of 156 randomly selected SNPs, and the corresponding empirical *P*-value

Gene list (number of genes)	Nearest		19 kb		94 kb		138 kb		258 kb	
	*N*	*P*	*N*	*P*	*N*	*P*	*N*	*P*	*N*	*P*
All genes (1362)	7	2.83E-01	5	6.13E-01	13	2.50E-01	25	4.43E-02	47	1.09E-02
Dominant (475)	3	3.28E-01	3	3.26E-01	3	5.58E-01	5	3.23E-01	10	1.37E-01
Recessive (936)	4	4.12E-01	2	8.60E-01	10	1.95E-01	20	2.00E-02	38	2.70E-03
Recessive excluding dominant (887)	4	3.70E-01	2	8.46E-01	10	1.68E-01	20	1.45E-02	37	1.40E-03

**Table 6:** Number of randomly selected SNPs (*N* = 156 SNPs) for which a DD gene is the nearest gene, or for which a DD gene is within the corresponding window, and the corresponding empirical *P*-value

Gene list (number of genes)	Nearest		19 kb		94 kb		138 kb		258 kb	
	*N*	*P*	*N*	*P*	*N*	*P*	*N*	*P*	*N*	*P*
All genes (1362)	7	9.44E-01	5	9.62E-01	14	8.87E-01	24	4.50E-01	35	5.40E-01
Dominant (475)	3	8.68E-01	3	6.94E-01	3	9.72E-01	5	9.53E-01	10	9.31E-01
Recessive (936)	4	9.36E-01	2	9.87E-01	11	7.24E-01	19	2.50E-01	29	2.26E-01
Recessive excluding dominant (887)	4	9.01E-01	2	9.82E-01	11	6.36E-01	19	1.74E-01	28	1.97E-01

### Birth weight SNPs

#### Nearest gene

The full list of genes linked to rare monogenic DDs contained 1362 autosomal genes. Of these, 20 were the closest gene for at least one lead SNP from the GWAS of birth weight. The *P*-value for enrichment compared with the empirical distribution of matching SNPs was *P* = 0.0002 (Table 7). Of the 156 birth weight SNPs, the nearest gene for 22 SNPs was in the full list of DD genes (*P* = 0.0036) (Table 8). When we split the list of genes into those that cause disease via either a dominant (*n* = 475) or recessive (*n* = 887) mode of inheritance only, 14 dominant genes were the nearest gene of at least one birth weight SNP (*P* < 0.0001) compared with six recessive-only genes (*P* = 0.17). The nearest gene for 15 of the birth weight SNPs was in the list of dominant DD genes (*P* < 0.0001), and by comparison, the nearest gene for only 7 of the birth weight SNPs was a DD gene with recessive-only effects (*P* = 0.55).

**Table 7:** Number of DD genes which are the nearest gene, or within the corresponding window, of 156 birth weight SNPs annotated as either ‘Fetal Only’, ‘Maternal and Fetal’ or ‘Unclassified’, and the corresponding empirical *P*-value

Gene list (number of genes)	Nearest		19 kb		94 kb		138 kb		258 kb	
	*N*_genes_	*P*	*N*_genes_	*P*	*N*_genes_	*P*	*N*_genes_	*P*	*N*_genes_	*P*
All genes (1362)	20	2.00E-04	22	2.10E-03	48	2.00E-04	57	1.50E-03	82	1.00E-03
Dominant (475)	14	<1.00E-4	13	<1.00E-4	25	<1.00E-4	27	<1.00E-4	36	3.00E-04
Recessive (936)	10	1.90E-02	13	1.90E-02	27	9.70E-03	35	7.50E-03	51	5.90E-03
Recessive excluding dominant (887)	6	1.68E-01	9	1.15E-01	23	2.48E-02	30	1.67E-02	46	1.02E-02

**Table 8:** Number of birth weight SNPs (total *N*_SNPs_ = 156) for which a DD gene is the nearest gene, or for which a DD gene is within the corresponding window, and the corresponding empirical *P*-value. Birth weight SNPs included are all those classified as classified as ‘Fetal Only’, ‘Maternal and Fetal’ or ‘Unclassified’ in the GWAS of birth weight (5)

Gene list (number of genes)	Nearest		19 kb		94 kb		138 kb		258 kb	
	*N*_SNPs_	*P*	*N*_SNPs_	*P*	*N*_SNPs_	*P*	*N*_SNPs_	*P*	*N*_SNPs_	*P*
All genes (1362)	22	3.60E-03	24	5.00E-03	45	1.20E-03	50	1.09E-02	67	1.12E-02
Dominant (475)	15	<1.00E-04	14	8.00E-04	26	<1.00E-04	27	1.30E-03	37	1.30E-03
Recessive (936)	12	8.37E-02	15	5.53E-02	29	6.65E-02	25	7.91E-02	48	4.42E-02
Recessive excluding dominant (887)	7	5.47E-01	10	4.18E-01	24	2.49E-01	29	3.35E-01	42	1.85E-01

**Table 9:** Dominant DD genes falling near to birth weight SNPs in each of our nearest gene or gene window analyses. Columns 5–9 indicate the number of birth weight SNPs for which that gene was the nearest gene or was within the relevant gene window

Gene name	Mode of inheritance	SNPs	SNP classifications	Nearest	19 kb	94 kb	138 kb	258 kb
*CCND2*	Dominant	rs76895963	Fetal and Maternal_-_Same Direction	1	1	1	1	1
*JAG1*	Dominant	rs6040076	Unclassified	1	1	1	1	1
*PIK3R1*	Dominant/recessive	rs28365970	Unclassified	1	1	1	1	1
*PTCH1*	Dominant/recessive	rs28457693	Fetal Only	1	1	1	1	1
*PTH1R*	Dominant	rs2168443	Fetal and Maternal_-_Same Direction	1	1	1	1	1
*RIT1*	Dominant	rs670523	Unclassified	1	1	1	1	1
*RORA*	Dominant	rs339969	Fetal and Maternal_-_Same Direction	1	1	1	1	1
*SPRED1*	Dominant	rs75844534	Fetal and Maternal_-_Opposite Directions	1	1	1	1	1
*STAT1*	Dominant/recessive	rs2280235	Unclassified	1	1	1	1	1
*WT1*	Dominant	rs5030317	Unclassified	1	1	1	1	1
*IGF2*	Dominant	rs11042596	Fetal Only	1		1	1	1
*PDE10A*	Dominant	rs2934844	Unclassified	1		1	1	1
*MAFB*	Dominant	rs1012167	Fetal Only	1				1
*IGF1R*	Dominant/recessive	rs11630479; rs7402983	Unclassified; Fetal Only	2	2	2	2	2
*NR2F2*	Dominant	rs55958435; rs138715366	Unclassified		1	1	1	1
*CAMK2B*	Dominant	rs2908279	Fetal Only; Unclassified		1	2	2	2
*CDKN1C*	Dominant	rs234864	Fetal Only			1	1	1
*CNOT3*	Dominant	rs255773	Unclassified			1	1	1
*DLG4*	Dominant	rs222857	Fetal Only			1	1	1
*EDNRB*	Dominant	rs9318511	Unclassified			1	1	1
*FGFR1*	Dominant	rs34036147	Fetal Only			1	1	1
*HIST1H1E*	Dominant	rs9379832	Unclassified			1	1	1
*HIST1H4C*	Dominant	rs9379832	Unclassified			1	1	1
*KIF11*	Dominant	rs1112718	Fetal and Maternal_-_Opposite Directions			1	1	1
*SLC2A1*	Dominant	rs12401656	Fetal Only			1	1	1
*P4HB*	Dominant	rs9912553; rs73354194	Unclassified; Fetal Only			1	1	2
*GJC2*	Dominant/recessive	rs708122	Unclassified				1	1
*SETD2*	Dominant	rs2168443	Fetal and Maternal_-_Same Direction				1	1
*ACVR1*	Dominant	rs56188432	Fetal Only					1
*CHD3*	Dominant	rs78378222	Unclassified					1
*GNAS*	Dominant	rs6026449	Fetal Only					1
*KAT6A*	Dominant	rs13266210	Fetal Only					1
*NF1*	Dominant	rs7223535	Unclassified					1
*NOTCH1*	Dominant	rs28505901	Fetal Only					1
*PHF21A*	Dominant	rs10437653	Unclassified					1
*PITX3*	Dominant	rs562974282	Fetal and Maternal_-_Opposite Directions					1

#### Gene windows

Of the full list of DD genes, 22, 48, 57 and 82 genes fell within 19, 94, 138 and 258 kb of at least one birth weight SNP, respectively (*P* = 0.0021; *P* = 0.0002; *P* = 0.0015; *P* = 0.001, respectively). Of the birth weight SNPs, 24, 45, 50 and 67 SNPs had at least one gene from the full gene list within 19, 94, 138 and 258 kb, respectively (*P* = 0.005; *P* = 0.0012; *P* = 0.011; *P* = 0.011). Genes in which rare mutations cause dominant DDs showed strong evidence of enrichment within the gene windows analysis with 13, 25, 27 and 36 genes, respectively, in the 19, 94, 138 and 258 kb windows (*P* < 0.0001; *P* < 0.0001; *P* < 0.0001; *P* = 0.0003). Of the birth weight SNPs, 14, 26, 27 and 37 had at least one dominant disease gene within each of the windows (*P* = 0.0008; *P* < 0.0001; *P* = 0.0013; *P* = 0.0013). There was little evidence that genes in which rare mutations cause only recessive disease showed any enrichment in the gene window analyses: a total of 9, 23, 30 and 46 (*P* = 0.12; *P* = 0.025; *P* = 0.017; *P* = 0.010) genes were within each window of at least 1 birth weight SNP, while a total of 10, 24, 29 and 42 (*P* = 0.42; *P* = 0.25; *P* = 0.34; *P* = 0.19) birth weight SNPs had at least one recessive-only gene within each window, respectively.

Results from sensitivity analysis excluding ‘Unclassified’ birth weight SNPs showed similar patterns (Supplementary Material, Tables S1 and S2), where evidence of enrichment was driven by genes with dominant modes of inheritance (all *P* < 0.05). There was little evidence of enrichment among genes with recessive-only effects (all *P* > 0.05). (Supplementary Material, Tables S3 and S4).

## Discussion

This is the first study to investigate the overlap between birth weight GWAS signals and genes known to cause rare DDs. We found that common lead SNPs from GWAS which are associated with birth weight, either partly or entirely through direct fetal effects, fall disproportionately closer to such genes than to randomly selected similar genes. This enrichment for associations was driven by DD genes with dominant modes of inheritance (Table 9), and the pattern was seen both for the nearest gene analysis and for all window sizes in the gene window analyses. We validated our method using height SNPs as positive controls which have previously shown enrichment for proximity to genes associated with rare human conditions of abnormal skeletal growth. Negative controls using random SNPs as well as those robustly associated with eye color show that these associations are unlikely to represent spurious associations.

The interpretation of GWAS loci and the genes and pathways impacted by them for complex traits such as birth weight is less straightforward than that of molecular phenotypes such as urate levels (23). Rare monogenic variants that cause severe disease are unlikely to underlie the associations with common SNPs that are identified in GWAS (24). Rather, the lead SNPs are far more likely to tag functional variants of a similar frequency. Genes that are causally linked with any phenotype may harbor a spectrum of genetic variants from rare with severe consequences (such as complete loss of gene function) to common with mild consequences (such as reduced gene expression). Our results support this hypothesis and show that the genes implicated in rare developmental syndromes can help to prioritize candidate causal genes at birth weight loci.

**Figure 2:**
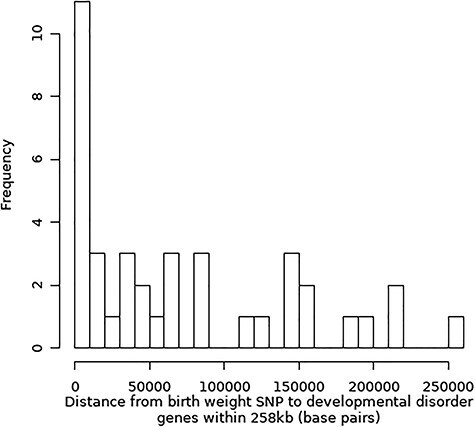
Histogram of the distance from birth weight SNPs to DD genes within 258 kb of the SNP.

Of the 37 birth weight SNPs with DD genes within the largest 258 kb window, this gene is the nearest one for just 13. A histogram of the distance from these SNPs to the DD gene is shown in Figure 2. While a DD gene is unlikely to be the relevant functional gene for every birth weight SNP, this result nonetheless highlights the fact that the nearest gene to the SNP is not necessarily the best candidate for functionally relevant genes. Our analysis has also helped to categorize GWAS SNPs that were previously unclassified with respect to maternal or fetal activity and to prioritize likely candidate genes. For example, high birth weight is a feature of Noonan syndrome, which can be caused by missense mutations in the *RIT1* gene (25); one of the birth weight SNPs, ‘Unclassified’ in the recent birth weight GWAS, lies within the gene boundaries of *RIT1*, suggesting that the SNP is acting through the fetal genome.

**Figure 3:**
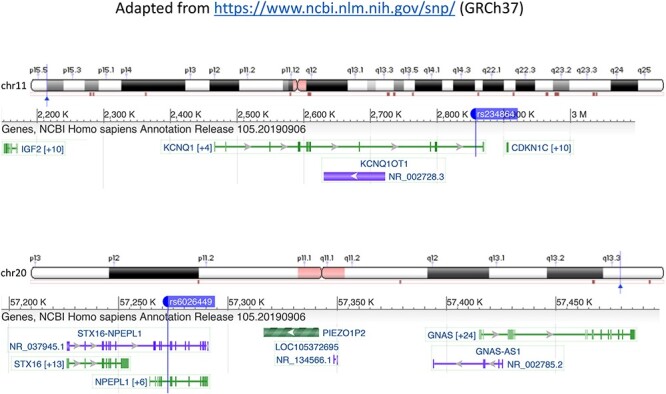
Regions surrounding the *CDKN1C* and *GNAS* genes showing the location of the genes and the nearby birth weight SNP. Colors represent different functional annotations and numbers in square brackets indicate additional transcripts.

Examples of DD genes whose associated syndromes include low or high birth weight, and which are nearby but not the nearest gene to the birth weight SNP, are *CDKN1C* and *GNAS*. *CDKN1C* is implicated in syndromes associated with intrauterine growth restriction (IUGR) (IMAGE syndrome) (26) and over-growth (Beckwith-Wiedemann syndrome) (27); one of the birth weight SNPs is located 47 146 bp from this gene within an intron of *KCNQ1*, which is not linked to DDs. Beckwith-Wiedemann syndrome can be caused by disorders of methylation affecting imprinted genes within chromosome 11p15.5 containing *IGF2* and *CDKN1C*, both of which appeared in our analyses (Fig. 3). *GNAS* has also been implicated in fetal growth, with mutations in the paternally inherited copy of the *GNAS* gene shown to lead to severe IUGR (28) and loss of methylation leading to increased fetal growth (29). Rare mutations in this gene are also linked with low birth weight in the DECIPHER database (30) (https://decipher.sanger.ac.uk/gene/GNAS#overview/clinical-info), but the closest birth weight SNP to *GNAS* is 142 178 bp away, within the *NPEPL1* gene (Fig. 3). These findings support the hypothesis that the nearest gene to a SNP identified via GWAS may not always be the biologically relevant gene (31). Syndromes resulting in large changes in birth weight associated with both of these DD genes also feature disorders of imprinting. Imprinted genes have previously been found to be enriched for birth weight associations (5), but so far, no parent-of-origin specific associations have been identified at individual loci. Our approach highlights these genes as potential candidates for identifying imprinting effects affecting birth weight within the normal range.

Candidate genes highlighted by our analyses can also point toward relevant biological pathways. For example, they include three genes that are linked by IGF-1 receptor signaling (*PIK3R1*, *IGF1R* and *IGF2*). Two of these genes (*PIK3R1* and *IGF1R*) have one and two birth weight lead SNPs within the boundaries of the genes, respectively, while the third gene (*IGF2*) is 31 481 bp from the nearest birth weight lead SNP. DDs caused by variation in each of these genes are all characterized by severe effects on fetal growth. Mutations in *PIK3R* are associated with the SHORT syndrome which is characterized by IUGR (32–34), and mutations causing dysregulation of *IGF1R* can also result in IUGR (35). The *IGF2* gene is implicated in syndromes associated with fetal under-growth (Silver-Russell syndrome) or over-growth (Beckwith-Wiedemann syndrome) (36). Furthermore, genes in the sonic hedgehog pathway have been implicated in the regulation of IGF-1 receptor signalling (37). Genes from this pathway, such as *PTCH1* and *GLI2*, appear in our DD gene list, but only *PTCH1* appears in proximity to a birth weight locus in any of our analyses. Rare mutations in *PTCH1* are associated with high birth weight in the DECIPHER database, and lower levels of *PTCH1* expression in preeclamptic placenta samples has been demonstrated, with strong associations between expression levels and birth weight (37). Enrichment for SNP associations with birth weight in pathways linked to these genes has previously been demonstrated (5), but our approach highlights individual genes within the pathway which may be particularly relevant to variation in birth weight.

A pathway which was not specifically highlighted in the recent GWAS of birth weight but has come up in our analysis is the Notch signaling pathway. Alagille syndrome, caused by rare mutations in *JAG1* and *NOTCH2*, includes failure to thrive (38) within its phenotypic spectrum. While it is not certain whether the birth weight associated SNP near *JAG1* acts primarily via fetal or maternal mechanisms, a reduced expression of *JAG1* in placentas from pregnancies complicated with preeclampsia has been observed (39). Although no association was seen between *JAG1* levels and birth weight, preeclampsia is itself associated with reduced birth weight. Other genes in the Notch pathway, *NOTCH1*, *NOTCH2*, *DLL3* and *DLL4*, were also included in our list of DD genes. Only one of these was highlighted in any of our analyses, *NOTCH1*, in the 258 kb gene windows analysis, whose expression level has not previously been linked with birth weight.

A large number of birth weight loci overlap with loci known to be associated with height. It is perhaps not surprising that several of the DD genes highlighted by our analysis are known to be associated with short stature, such as *IGF1R*, *IGF2*, *RIT1* and *NF1*.

In the present study, we have described a method for combining information from common and rare disease genetics to help prioritize candidate genes through which GWAS loci may act. We were limited by several factors. First, the list of monogenic genes we used was clinically curated as part of the Deciphering Developmental Disorders Study (18) and we included any genes implicated in DDs, some of which are well known to cause extremes of birth weight while others do not have birth weight recorded as a feature of the associated disorder. The inclusion of genes without effects on birth weight could reduce the power of the analysis to detect associations owing to the inclusion of irrelevant genes. We nonetheless chose to include these genes owing to the extreme rarity of many of the disorders, and thus the limited availability of detailed phenotypes. Had we excluded all genes that did not have birth weight recorded as a feature of the associated disorder, we would likely have excluded genes with effects on birth weight, that were simply not recorded due to incomplete phenotyping, which would similarly reduce power. Second, the list of birth weight loci also included those categorized as ‘Unclassified’, some of which are likely to act solely through maternal pathways. Accurate classification of these loci would also increase the power to detect enrichment, though the results of our sensitivity analysis where these loci were excluded, while less powered, were consistent with the main analysis. Third, while we performed negative control analysis using SNPs known to influence eye color, only 16 eye color-associated SNPs were available. Eye color was chosen as a control trait because it is unlikely to be associated with DDs, but for future application of the method, a control trait with a larger number of associated SNPs would be beneficial.

In summary, we have described a newly developed method and software package for testing GWAS loci for the enrichment for proximity to genes implicated in monogenic disorders and have demonstrated an enrichment in birth weight GWAS loci with fetal effects for proximity to genes where rare mutations are known to cause DDs. This method could help prioritize candidate variants from other GWAS to help better understand the mechanisms underlying their phenotypic effect.

## Materials and Methods

### Birth weight SNPs

We selected the lead SNP at each of the 190 genomic loci (*P* < 6.6 × 10^−9^ and *r*^2^ < 0.1) from the latest GWAS of birth weight (5). Where a locus was known to have different lead SNPs from the maternal GWAS of offspring birth weight versus the GWAS of own birth weight (‘fetal GWAS’), we selected the lead SNP from the fetal GWAS. In that study, the 190 loci had been classified into categories according to the likely origin of their effects on birth weight (Supplementary Material, Table S5): ‘Fetal only’ (62 SNPs); ‘Maternal only’ (31 SNPs); ‘Fetal and Maternal’ (35 SNPs) and ‘Unclassified’ (62 SNPs). SNPs are unclassified if the 95% confidence intervals for independent maternal and fetal effect estimates overlap, and at least one overlaps zero (5). Since we were interested in investigating loci with the direct fetal effects on birth weight, we excluded the loci classified as ‘Maternal only’ from our analyses. Loci on chromosome X (*N* = 4) were also excluded from our analyses owing to the difficulty in classifying X-chromosome genes as dominant or recessive. The resulting list of lead SNPs used in our analysis included 156 SNPs.

### Positive control: height SNPs

Growth abnormalities and extreme variation in height are a core feature of many DDs, and height GWAS SNPs have previously been shown to be enriched for genes known to be involved in growth (17). As a positive control, we tested whether our method showed evidence of enrichment for the height associated SNPs identified by Wood *et al*. (17). Of the 697 identified by Wood *et al*., we used the 694 SNPs appearing in our list of UK Biobank Haplotype Reference Consortium (HRC) imputed SNPs (detailed later) (Supplementary Material, Table S6).

### Negative controls: eye color and random SNPs

To further test the validity of our method, we performed negative control analyses where we would not expect to see strong evidence of enrichment for proximity to DD genes. First, we reasoned that SNPs associated with eye color would not be expected to fall closer to DD genes than would be expected by chance, so we tested enrichment for proximity to DD genes of 16 SNPs associated with eye color at *P* < 5 × 10^−8^ (19) to use as a negative control analysis (Supplementary Material, Table S7). Second, owing to the limited number of SNPs associated with eye color, we further selected 156 SNPs randomly from our list of HRC SNPs (see in the following text).

### Gene lists

A clinician-curated list of protein-coding genes definitively linked to monogenic disorders (20) was downloaded from https://www.ebi.ac.uk/gene2phenotype/ on 18 July 2018. Genes on the X-chromosome were excluded. Genes were separated into groups based on the mode of inheritance of their associated DDs (dominant, recessive or both). The list of DD genes can be found in Supplementary Material, Table S8.

### Enrichment analysis

We aimed to test whether our 156 selected lead SNPs, marking common fetal variant effects on birth weight, fall near to genes in which rare variants cause DDs (that may include high or low birth weight) more often than would be expected by chance, i.e. we tested for ‘enrichment’ of proximity to developmental syndrome genes in our list of GWAS SNPs. For each enrichment analysis, we used the 17 073 342 SNPs with minor allele frequency (MAF) > = 0.1% included in the UK Biobank HRC imputed dataset (release v3 March 2018) as a reference (21). From this list of reference SNPs, we selected 10 000 lists of SNPs, which were matched to the lead SNPs from GWAS of birth weight based on the matching criteria listed in the following text. These lists of matching SNPs were used to create an empirical distribution, described later, from which we calculated empirical *P*-values for the corresponding list of birth weight loci (Fig. 1). We used two sets of matching criteria: (1) the distance to the nearest gene and (2) the number of genes within a given distance. We repeated these analyses splitting the list of DD genes into those with dominant modes of inheritance and those with recessive modes of inheritance. These criteria are described in more detail in the following sections, and the code required to run the analysis has been packaged and can be downloaded from https://github.com/rnbeaumont/DD_gene_enrichment.

#### Nearest gene

Each of the 156 birth weight lead SNPs was annotated with its nearest gene and the distance to that gene. The criteria for selecting 10 000 lists of matched SNPs for the nearest gene analysis were: MAF for the matching SNP between 0.9 and 1.1× the MAF of the index SNP; and distance to the nearest gene of the matching SNP within ±10% of the distance of the index SNP to the nearest gene. For each of the 10 000 lists of matched SNPs, we calculated the number of SNPs for which their nearest gene appeared in the lists of DD genes. We also calculated the number of DD genes that appeared in the nearest gene list for the matched SNPs. These were used as our empirical distributions. We then calculated the number of nearest genes for the birth weight loci which appeared in the DD genes lists and vice versa.

#### Gene windows

We annotated each birth weight SNP with the number of genes 19, 94, 138 and 256 kb either side of the SNP. To select window sizes objectively, we chose the mean, median, lower quartile and upper quartile of the distances from lead birth weight SNPs to eight placenta eQTL genes (22) from the Warrington *et al*. GWAS of birth weight (5) as these represent biologically plausible distances between functional units, although our results are unlikely to be sensitive to the exact window sizes used. The criteria for selecting the 10 000 lists of matched SNPs for the gene window analyses were: the MAF of the matching SNP within 0.9–1.1× the MAF of the lead birth weight SNP; and number of genes within the window matching that of the lead birth weight SNP. For each list of the lists of matching SNPs, we calculated the number of SNPs for which one or more of the genes within the relevant window were in the list of DD genes and the number of those genes which appear within the relevant distance of at least one matched SNP. The empirical *P*-values for the number of birth weight SNPs for which at least one of the genes within the window appear in the list of DD genes and vice versa using the empirical distributions.

### Sensitivity analysis

These analyses were repeated excluding birth weight SNPs categorized as ‘Unclassified’ (5) as a sensitivity analysis, as that category could include SNPs with maternal effects.

## Supplementary Material

Supplemental_Tables_ddab060

## Data Availability

Data used in these analyses is publicly available: birth weight summary statistics can be downloaded from http://egg-consortium.org/; the list of HRC SNPs can be downloaded from http://www.haplotype-reference-consortium.org/site; access information for UK Biobank can be found at https://www.ukbiobank.ac.uk/; a list of DDD genes can be downloaded from https://www.ebi.ac.uk/gene2phenotype/ (accessed 18th July 2018). Positive control data was downloaded from https://portals.broadinstitute.org/collaboration/giant/index.php/GIANT_consortium_data_files (accessed 14 October 2020), and negative control data was downloaded from https://www.ebi.ac.uk/gwas/efotraits/EFO_0009764 (accessed 2 October 2020). Code to run the analysis can be found on github at https://github.com/rnbeaumont/DD_gene_enrichment.
